# Unfamiliar partnerships limit cnidarian holobiont acclimation to warming

**DOI:** 10.1111/gcb.15263

**Published:** 2020-07-26

**Authors:** Marcela Herrera, Shannon G. Klein, Sebastian Schmidt‐Roach, Sara Campana, Maha J. Cziesielski, Jit Ern Chen, Carlos M. Duarte, Manuel Aranda

**Affiliations:** ^1^ Red Sea Research Center (RSRC), Biological and Environmental Sciences & Engineering Division (BESE) King Abdullah University of Science and Technology (KAUST) Thuwal Saudi Arabia; ^2^ Red Sea Research Center (RSRC) and Computational Bioscience Research Center (CBRC), Biological and Environmental Sciences & Engineering Division (BESE) King Abdullah University of Science and Technology (KAUST) Thuwal Saudi Arabia; ^3^ Present address: Faculty of Science Institute for Biodiversity and Ecosystem Dynamics University of Amsterdam 1090 GE Amsterdam The Netherlands; ^4^ Present address: School of Science and Technology Department of Biological Sciences Sunway University Subang Jaya Selangor Malaysia

**Keywords:** adaptation, climate change, coral reefs, *Exaiptasia pallida*, heat stress, photosynthesis, respiration, Symbiodiniaceae, SymPortal

## Abstract

Enhancing the resilience of corals to rising temperatures is now a matter of urgency, leading to growing efforts to explore the use of heat tolerant symbiont species to improve their thermal resilience. The notion that adaptive traits can be retained by transferring the symbionts alone, however, challenges the holobiont concept, a fundamental paradigm in coral research. Holobiont traits are products of a specific community (holobiont) and all its co‐evolutionary and local adaptations, which might limit the retention or transference of holobiont traits by exchanging only one partner. Here we evaluate how interchanging partners affect the short‐ and long‐term performance of holobionts under heat stress using clonal lineages of the cnidarian model system Aiptasia (host and Symbiodiniaceae strains) originating from distinct thermal environments. Our results show that holobionts from more thermally variable environments have higher plasticity to heat stress, but this resilience could not be transferred to other host genotypes through the exchange of symbionts. Importantly, our findings highlight the role of the host in determining holobiont productivity in response to thermal stress and indicate that local adaptations of holobionts will likely limit the efficacy of interchanging unfamiliar compartments to enhance thermal tolerance.

## Graphical abstract

The cnidarian holobiont is a distinct, interactive biological entity comprised of three main components: the animal host, algal symbionts of the family Symbiodiniaceae and an extensive microbiota. Holobiont traits, such as thermal resilience, are thus a product of a specific species assemblage and therefore not necessarily retained when only one partner is exchanged (holobionts iii and iv). Consequently, non‐native associations tend to perform at a suboptimal level (here shown as puzzle pieces that do not fit together).

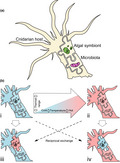

## 1 INTRODUCTION

The interaction between animals and dinoflagellates of the family Symbiodiniaceae is one of the most abundant, widespread, and ecologically successful symbioses found in nature (Kirk & Weis, 2016). Numerous taxa, ranging from protists and sponges to cnidarians, flatworms, and mollusks (Kirk & Weis, 2016; Decelle et al., 2018) benefit from this symbiotic association. For shallow‐water corals—the foundation species of coral reefs—this relationship can be highly obligate such that its breakdown (i.e., bleaching) often results in death (Hoegh‐Guldberg, 1999). Thus, the maintenance of this partnership is vital for the continual growth and survival of entire ecosystems.

Warming sea surface temperatures, as a consequence of climate change, are causing more frequent and severe mass bleaching events worldwide that precipitate the global decline of coral reefs (Hoegh‐Guldberg, Poloczanska, Skirving, & Dove, 2017). Predictions reveal that current trends of global warming will result in a further loss of up to 90% compared to today (Masson‐Delmotte et al., 2018; van Hooidonk et al., 2016). In light of this, efforts to enhance corals’ resilience have accelerated (van Oppen et al., 2017; van Oppen, Oliver, Putnam, & Gates, 2015), including microbiome engineering (Damjanovic, Blackall, Webster, & van Oppen, 2017; Epstein, Smith, Torda, & van Oppen, 2019; Rosado et al., 2019). Indeed, there is ample evidence for the importance of microbes in the adaptive responses of corals to changes in the environment. Composition of their Symbiodiniaceae communities may vary before, during, and after stress exposure (i.e., symbiont shuffling and/or switching; Baker, 2003; Boulotte et al., 2016).

**FIGURE 1:**
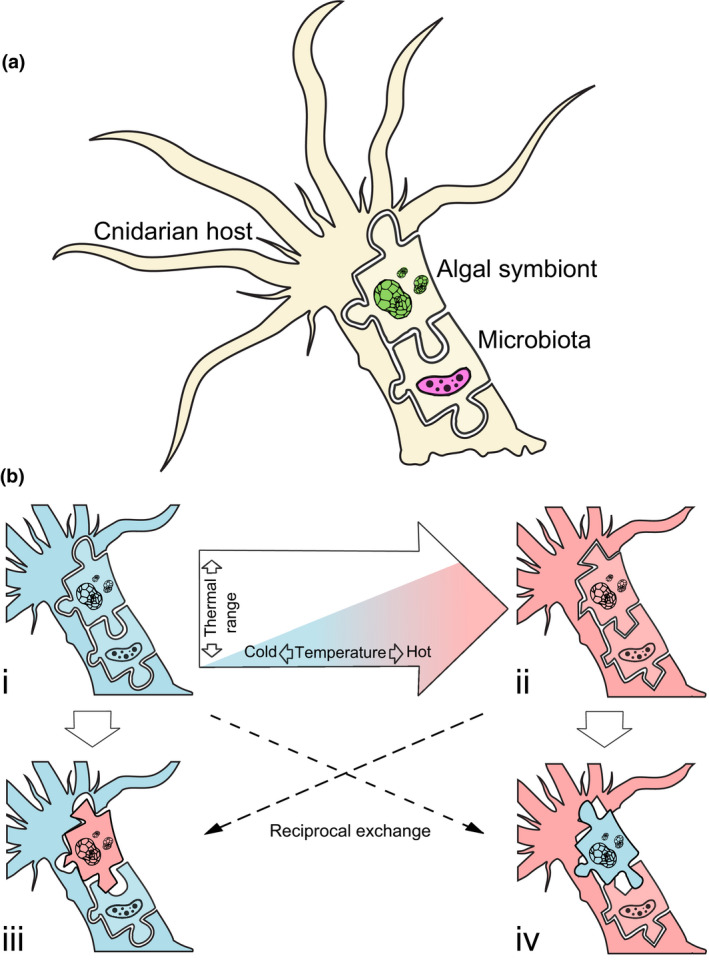
(a) The cnidarian holobiont is a distinct, interactive biological entity comprised by three main components: the animal host, algal symbionts of the family Symbiodiniaceae, and an extensive microbiota including bacteria, viruses, archaea, fungi, endolithic algae, and protists. (b) Holobiont traits (i.e., thermal resilience of holobionts i and ii) are not necessarily retained when translocating only one partner (holobionts iii and iv). Instead, non‐native associations tend to perform at a suboptimal level (here shown as puzzle pieces that do not fit together)

Certain symbiont species have been shown to increase heat tolerance of corals by up to 1.5°C (Berkelmans & van Oppen, 2006). Thus, it has been proposed that interchanging symbionts (and also hosts) with more resistant types might be a promising way to increase the thermal resilience of cnidarian holobionts (Chakravarti, Beltran, & van Oppen, 2017; Coles & Riegl, 2013; Cunning, Silverstein, & Baker, 2018; McIlroy et al., 2016; Morikawa & Palumbi, 2019; Palumbi, Barshis, Traylor‐Knowles, & Bay, 2014; Thomas et al., 2018; Figure 1). These approaches, however, conflict with the growing body of work that points toward the emergent properties of a holobiont system being “larger than the sum of its parts” (Bordenstein & Theis, 2015; Dittami et al., 2019; Rosenberg & Zilber‐Rosenberg, 2018). The host with its microbiota (Figure 1)—the holobiont—is without doubt a distinct, interactive biological entity, and ultimately can be, in many instances, a unit of selection (Rosenberg, Sharon, Atad, & Zilber‐Rosenberg, 2010; Rosenberg, Sharon, & Zilber‐Rosenberg, 2009; Rosenberg & Zilber‐Rosenberg, 2018; but see Douglas & Werren, 2016; Moran & Sloan, 2015). As holobionts constitute functional aggregates that are “interactors,” “reproducers,” and “manifestors of adaptation” (Roughgarden, Gilbert, Rosenberg, Zilber‐Rosenberg, & Lloyd, 2018), studying their closely intertwined ecology and evolution is uniquely challenging, especially in the context of environmental change. Present‐day corals have already, and will continue to experience, rapid changes in their thermal environment and must undergo short‐term adaptation, such as changes in their microbial communities toward more resilient types, to ensure their persistence. Novel (more resistant) symbioses are temporally limited and rarely persist over ecological timescales (LaJeunesse, Smith, Finney, & Oxenford, 2009; Thornhill, LaJeunesse, Kemp, Fitt, & Schmidt, 2006), thus highlighting the complex nature (metabolically, immunologically, developmental, etc.) of symbiotic associations. The viability of transferring more resilient symbiont types as a means of improving holobiont resilience is likely contingent on the long‐term evolution of the holobiont compartments (Figure 1) and yet, the level of importance given to this consideration is debatable.

However, we still do not understand to what extent holobiont traits are products of co‐evolutionary processes and local adaptions that also underlie host–symbiont fidelity and performance, making them specific to a given host–symbiont combination and environment. Most studies focusing on understanding the cellular and physiological drivers that limit the capacity for novel symbioses to arise have, however, used foreign, non‐native photosymbiont species (Gabay, Parkinson, Wilkinson, Weis, & Davy, 2019; Gabay, Weis, & Davy, 2018; Goulet, Cook, & Goulet, 2005; Starzak, Quinnell, Nitschke, & Davy, 2014; Ye, Bhattacharjee, & Siemann, 2019). Here we built on these studies by using symbiont strains that are naturally found in the same host, and from different climatic regions of the host's distribution range. This approach allows us to test if it is possible to retain adaptations underlying host–symbiont specificity while increasing thermal resilience of hosts from colder regions. Furthermore, by testing the thermal performance of the different host–symbiont combinations, we can ascertain to what extent the partners contribute to thermal resilience and, therefore, their role in determining holobiont performance.

We examined the holobiont concept and the underlying complexity of host–microbe associations through time by actively manipulating the symbiotic communities of the sea anemone *Exaiptasia pallida* (Grajales & Rodríguez, 2014; hereafter referred to as “Aiptasia”). Aiptasia has been a model system for coral research for over 30 years (Baumgarten et al., 2015; Weis, Davy, Hoegh‐Guldberg, Rodriguez‐Lanetty, & Pringle, 2008) as it allows overcoming many of the limitations associated with corals. Aiptasia is a globally distributed species that can be bleached and colonized by different Symbiodiniaceae strains, facilitating the disentanglement of host and symbiont type effects (Gabay et al., 2018, 2019; Hambleton, Guse, & Pringle, 2014; Rädecker et al., 2018; Starzak et al., 2014; Wolfowicz et al., 2016). We used clonal lines from three different geographic locations with distinct temperature regimes: H2 from Hawaii (Xiang, Hambleton, DeNofrio, Pringle, & Grossman, 2013), CC7 from North Carolina (Sunagawa et al., 2009), and RS from the central Red Sea (Cziesielski et al., 2018). These lineages have almost certainly acquired specific genotypic adaptations to their local environment and differ in their natively associated symbionts (*Breviolum minutum*: Xiang et al., 2013; *Symbiodinium linucheae*: Bieri, Onishi, Xiang, Grossman, & Pringle, 2016; *Symbiodinium microadriaticum*: Cziesielski et al., 2018), thus resulting in different responses of both partners (Cziesielski et al., 2018). Red Sea Aiptasia may provide critical insight into mechanisms promoting the acclimatory capacity of symbiotic cnidarians due to their high thermal tolerance and ability to thrive in one of the warmest (as high as 35°C during summer; Ngugi, Antunes, Brune, & Stingl, 2012) seas on Earth (Berumen et al., 2019). Based on the premise that host and symbiont species from the Red Sea are more heat tolerant, we studied their contribution to the thermal resilience of Aiptasia holobionts that originate from lower temperature environments. Furthermore, we tested whether specific adaptations of the different Aiptasia‐Symbiodiniaceae combinations might limit the exchangeability of partners in novel symbioses. For this, we inoculated symbiont‐free polyps of all host lines with each pairwise combination of symbiont taxa and examined their functional variability under short‐ and long‐term thermal stress.

## 2 MATERIALS AND METHODS

### 2.1 Experimental set‐up: Aiptasia rearing, bleaching, and inoculations

Aiptasia from three different clonal laboratory strains were used in this study: H2 from Hawaii, CC7 from North Carolina and a Red Sea (RS) line obtained from the central coast of Saudi Arabia. H2’s homologous symbionts are *B. minutum* (Xiang et al., 2013; referred to as SSB01), whereas CC7 naturally associates with *S. linucheae* (Bieri et al., 2016; referred to as SSA01) but can also form stable symbioses with other species (Thornhill, Xiang, Pettay, Zhong, & Santos, 2013). Red Sea Aiptasia, on the other hand, occurs with both *S. microadriaticum* and *Breviolum* taxa (Cziesielski et al., 2018; Thornhill et al., 2013; here referred to as RS‐A and RS‐B, respectively; see Table 1). It is worth clarifying that all the symbiont strains we tested here are native to Aiptasia, yet, they are specific to each genotype so that homologous symbionts are those that regularly associate with a particular host and heterologous symbionts are those that typically do not associate with a given host. For example, while *B. minutum* is the homologous symbiont of H2‐Hawaii, it is a heterologous symbiont in CC7‐North Carolina.

**TABLE 1:** Details of the Symbiodiniaceae cultures used to perform inoculations. Name of the culture followed by the host source, original geographic location, Symbiodiniaceae species, and majority ITS2 sequence as determined by SymPortal

Symbiont culture	Aiptasia host source	Original geographic location	Symbiodiniaceae species	Majority ITS2 sequence
SSB01	H2	Hawaii, USA	*Breviolum minutum*	B1
SSA01	CC7	North Carolina, USA	*Symbiodinium linucheae*	A4
RS‐A	RS	Al Lith, Saudi Arabia (central Red Sea)	*Symbiodinium microadriaticum*	A1
RS‐B	RS	Al Lith, Saudi Arabia (central Red Sea)	*Breviolum* sp.	RS‐B1^a^

^a^ SymPortal revealed different ITS2 type profiles for the *Breviolum* strains from Hawaii and the Red Sea, thus these were classified as B1 and RS‐B1, respectively.

Anemones were reared in autoclaved natural seawater (~39 psu and pH ~ 8) at 25°C under ~40 μmol photons m^−2^ s^−1^ white light on a 12:12 hr light:dark cycle (daytime of 06:00–18:00). These light levels, which are similar to those reported by other studies working with Aiptasia (Cui et al., 2019; Gegner et al., 2017; Lehnert et al., 2014; Röthig et al., 2016), were chosen to support optimal growth of the animals but also because all symbiont strains used here perform well under this irradiance. Individuals were fed twice per week with freshly hatched *Artemia* brine shrimp. All populations were kept in Percival incubators (Model I‐22LLVL, Percival Scientific) under identical conditions. Menthol‐induced bleaching (Matthews et al., 2016) was used to generate aposymbiotic individuals of each clonal line. Animals were incubated in autoclaved seawater with 0.19 mmol/L menthol during daytime, followed by a 5 μmol/L 3‐(3,4‐dichlorophenyl)‐1,1‐dimethylurea seawater incubation overnight. This treatment was repeated until complete bleaching was observed and confirmed via fluorescence microscopy. Anemones were then kept in a dark incubator for at least 2 months and further maintained for at least another month on a diurnal 12:12 hr light:dark cycle to ensure there was no re‐establishment of symbiosis.

Cultures of the strains SSA01 and SSB01 (courtesy of the John Pringle Lab) were used to perform inoculations with *Symbiodinium* and *Breviolum* taxa, respectively. Furthermore, Symbiodiniaceae from the Red Sea line were isolated. Briefly, one anemone was washed with 500 µl of f/2 medium + K/A/S (Kanamycin/Ampicillin/Streptomycin: 50/100/50 µg/ml) and then crushed in 500 µl of fresh f/2 medium + K/A/S using a glass tissue grinder (Duran Wheaton Kimble) to keep the algal cells intact. This was filtered through a 40 µm nylon mesh sterile cell strainer (Fisherbrand, Fisher Scientific), diluted in 15 ml of f/2 medium + K/A/S and subsequently transferred to liquid culture flasks (225 cm^2^ Nunc Cell Culture Treated EasYFlask, Thermo Scientific) with 5 ml of the diluted fresh extract and 150 ml of f/2 medium + K/A/S + GeO_2_ (4.47 µg/ml) saturated solution was added to prevent diatom and bacteria growth. Presence of algal cells was checked under a fluorescence microscope (Leica DMI3000 B inverted phase contrast microscope, Leica Microsystems GmbH). Moreover, solid agar f/2 + K/A/S plates were inoculated with the same fresh extract (normal concentration, 5‐fold and 25‐fold dilution) so that single colonies could be grown and isolated for further experiments. After 10 days, 15–20 single colonies were picked from each plate, grown in 1.5 ml Eppendorf tubes with 300 µl of f/2 medium + K/A/S and transferred to culture flasks (75 cm^2^ Nunc Cell Culture Treated EasYFlask, Thermo Scientific), as previously described. The K/A/S treatment was performed for every 10 subcultures to clear the bacterial load, and verified by growth on marine broth and inspected under the microscope. The above was performed under a flow hood (NuAire) to avoid environmental contamination. All liquid and solid cultures were kept in an incubator (Model I‐22LLVL, Percival Scientific) at 29°C on a 12:12 hr light:dark cycle (80–100 µmol photons m^−2^ s^−1^ of photosynthetically active radiation).

Inoculations were performed so that six heterologous host–symbiont combinations were obtained: H2‐Hawaii + SSA01, H2‐Hawaii + RS, CC7‐North Carolina + SSB01, CC7‐North Carolina + RS, RS‐Red Sea + SSA01, and RS‐Red Sea + SSB01. Algal strains were grown in cultures for more than 1 year before conducting experiments. Cell density of each culture was assessed 1 day prior by flow cytometry (BD LSRFortessa, BD Biosciences) to accurately calculate the desired amount of 10^5^ cells/ml to perform the inoculations. In a flow hood, each culture flask was mixed well, and an aliquot was transferred to a 50 ml Falcon tube. The liquid was centrifuged for 5 min at 3,000 rcf, f/2 medium was removed and cells were resuspended in 30 ml of autoclaved seawater. The cell suspension was then poured in each 250 ml tank containing the aposymbiotic anemones, followed by immediate feeding with *Artemia* to facilitate the dinoflagellate uptake. Inoculations were verified by fluorescence microscopy. Individual anemones were inspected every day for the first 2 weeks until color pigmentation was visible to the naked eye. Colonization patterns (i.e., cell densities) in Aiptasia are generally stable after 12 weeks (Matthews et al., 2016); yet, inoculated animals were maintained (under the same conditions as described above) for at least 6 months before performing the following experiments.

### 2.2 Physiological performance under acute thermal stress

Fifteen individuals from each of the nine different Aiptasia host–symbiont combinations were subjected to acute heat stress as follows. Temperature was slowly ramped up starting at 25°C to 32.5°C with increments of 2.5°C every 1.5 hr, starting from 08:00 and reaching the target temperature by the afternoon (14:00). Anemones were transferred into 5 ml custom glass chambers fitted with an internal stir bar (chamber design by Dr. Julia Strahl, University of Oldenburg) and a FireSting O_2_ fiber‐optical oxygen sensor (PyroScience). Chambers were filled with autoclaved seawater and submerged in a 25°C water bath and maintained in darkness for 30 min to acclimate with the magnetic stirrers switched on to prevent stratification of the water column. O_2_ fluxes (μmol/L) were then recorded once every 15 s over the course of a 30 min incubation period in light (~40 μmol photons m^−2^ s^−1^), followed by 10 min acclimation in darkness (<1 μmol photons m^−2^ s^−1^) and then a 30 min incubation in dark. Temperature was increased by heating up the water tub and constantly monitoring it with a temperature probe. A 10 min acclimation time was allowed between each temperature increment to ensure the chamber had reached the target temperature. The above was repeated until a final temperature of 32.5°C was reached. Individuals were tested in different batches across several days so natural biological variation could be taken into account.

### 2.3 Gross photosynthesis, respiration, and P:R ratios

Net photosynthesis (P_net_) and respiration (R) rates were calculated from the slope of the linear increase and decrease in dissolved oxygen concentration during light and dark incubations, respectively, with each temperature increment. For every respirometry assay performed, values of P_net_ and R were corrected for background microbial oxygen consumption (i.e., seawater controls) and transformed into their carbon equivalents by assuming quotients of 1.1 and 0.9 (over 24 hr), respectively (Muscatine, McCloskey, & Marian, 1981). These were then normalized to symbiont counts (see below) in order to generate values of gross photosynthesis (P_gross_), expressed as pmol C cell^−1 ^hr^−1^. Photosynthesis to respiration ratios (P:R) were calculated (P_gross_/R, where P_gross_ = P_net_ + |R|), as an indicator of the autotrophic capacity.

### 2.4 Thermal response under long‐term stress

This experiment was conducted ~6 months after performing the respirometry assays. Ten Aiptasia from each of the host–symbiont combinations above were subjected to long‐term heat stress as follows. In brief, temperature was gradually ramped up from 25°C to 32°C over the course of 8 hr at increments of 1°C per hour as previously described (Gegner et al., 2017). A subbleaching temperature of 32°C was chosen for this experiment (Aiptasia at 39 psu salinity can resist up to 34°C without bleaching according to Gegner et al., 2017) in order to investigate the thermal response (see below) among the different host–symbiont combinations. Anemones remained at this temperature for 28 days, during which daily maximum photochemical efficiency of PSII (*Fv*/*Fm*) was recorded with a Pulse Amplitude Modulated fluorometer (Mini‐PAM, Walz) to assess the photophysiological status of each individual. Polyps were dark acclimated for 30 min prior to measurements. Of note, all remaining individuals of the H2‐Hawaii + RS combination died within a few weeks following the previous experiment. Furthermore, homologous RS‐Red Sea Aiptasia was not available at the time we performed this test.

### 2.5 Activation energy effect sizes

The thermal dependence of the processes measured above was parameterized as the activation energy (*E*_a_), expressed in electronvolts (eV). This was calculated in an equivalent manner to an effect size per unit temperature so it could be used to compare the magnitude of responses across multiple biological traits (Marbà, Jorda, Agusti, Girard, & Duarte, 2015):

$$E_{a}=\frac{\ln\frac{V_{0}}{V_{i}}}{\frac{1}{kT_{i}}‐\frac{1}{kT_{0}}},$$

where *V*_0_ is the value of the response variable observed previous to a thermal anomaly (*V*_i_) measured for temperatures (in Kelvin) *T*_0_ and *T*_i_, respectively, multiplied by the Boltzmann constant (*k* = 8.617734 × 10^–5^ eV; Regaudie‐de‐Gioux & Duarte, 2012), following the Arrhenius model (Dell, Pawar, & Savage, 2011). Here, activation energies were calculated as the ratio of the response variable at 25°C (*V*_0_) and 32.5°C (*V*_i_). *E*_a_ values <0 (i.e., negative values) indicate a decrease in the trait response with increasing temperature. *E*_a_ values ≈0 indicate no change in the trait response with increasing temperature, whereas *E*_a_ values >0 (i.e., positive values) indicate an increase in the trait response with warming.

### 2.6 DNA extraction, protein content, and symbiont cell counts

Genomic DNA for Symbiodiniaceae typing was isolated from 10 ml of liquid culture that were centrifuged at 1,500 rcf for 10 min, where the supernatant was removed and the pellet was resuspended in 0.5 ml of cell lysis buffer from the DNeasy Plant Mini Kit (Qiagen) in a 2 ml screw top Eppendorf tube. The cells were then homogenized using glass beads in a TissueLyser II (Qiagen) set at 30 Hz for 30 s. The homogenate was spun down at 16,000 rcf for 10 s and the supernatant was taken for all downstream processing steps, according to the manufacturer's instruction manual for the DNeasy Plant Mini Kit. This was done for each strain, from which three technical replicates were independently DNA‐extracted. Thus, each Symbiodiniaceae strain in this study is represented by only one culture line.

For Aiptasia samples, a tentacle from each individual was plucked to extract DNA with the Chelex 100^®^ (Bio‐Rad) resin method (Walsh, Metzger, & Higuchi, 1991). The tissue was vortexed with Chelex slurry for 20 s and briefly spun down in a picofuge. Samples were incubated for 20 min at 99°C, vortexed again for 20 s and spun down at 16,000 rcf for 2 min. Supernatant was later used as the template for PCR. Each anemone was then crushed in 500 µl of cell lysis buffer (200 mM TRIS pH 7.5, 2 M NaCl, 0.1% Triton 20%) and two aliquots of 100 and 400 µl were immediately snap‐frozen in liquid nitrogen and stored in −20°C for further protein content and symbiont concentration analysis, respectively. Total host protein content was quantified with a Micro BCA Protein Assay Kit (Thermo Scientific) using triplicates of 150 µl of 15×‐diluted tissue slurry as per manufacturer instructions. Protein concentrations were measured at 562 nm absorbance using a SpectraMax Paradigm Multi‐Mode Detection Platform (Molecular Devices). Symbiont cell counts were done by flow cytometry (BD LSRFortessa, BD Biosciences). To do this, tissue homogenate was spun down at 14,000 rcf for 5 min, supernatant was removed, and the pellet was resuspended in phosphate buffered saline solution. The latter was performed twice before filtering it through a 40 µm nylon mesh sterile cell strainer (Fisherbrand, Fisher Scientific). Cells were excited at a wavelength of 488 nm and fluorescence emission was recorded at 695/40 nm. Symbiont densities were quantified in triplicate measurements (20 μl each) based on forward‐scattered light and chlorophyll autofluorescence signals of recorded events.

### 2.7 ITS2 amplicon sequencing

Illumina sequencing of the Internal Transcriber Space 2 (ITS2) region was used to examine the Symbiodiniaceae community composition of all host–symbiont combinations. PCRs were performed in triplicates using the primers (Illumina adapters underlined below) SYM_VAR_5.82S2 (5′TCGTCGGCAGCGTCAGATGTGTATAAGAGACAG‐GAATTGCAGAACTCCGTGAACC3′) and SYM_VAR_REV (5′GTCTCGTGGGCTCGGAGATGTGTATAAGAGACAG‐CGGGTTCWCTTGTYTGACTTCATGC3′; Hume et al., 2018). Each PCR reaction was run with a Qiagen Multiplex PCR Kit (Qiagen) and 10 μM primers in a final reaction volume of 15 μl. Thermal cycling conditions of 15 min at 95°C, followed by 30 cycles of 30 s at 95°C, 90 s at 56°C, and 30 s at 72°C, with a final extension step of 10 min at 72°C were used for amplification. For each sample, PCR products were run on a 1% agarose electrophoresis gel, pooled and cleaned using ExoProStar 1‐step (GE Healthcare). Indexing was then performed using the Nextera XT Index Kit (Illumina) according to the manufacturer's instructions followed by sample normalization and final library pooling. A SequalPrep Normalization Plate Kit (Invitrogen, Thermo Fisher Scientific) was used to do the normalization, avoiding the more labor‐intensive process of quantifying and aliquoting each individual sample. The final pooled library was quantified on a BioAnalyzer (Agilent Technologies) and sequenced at 7 pM with 20% phiX on the Illumina MiSeq, 2 × 300 bp end version 3 chemistry according to the manufacturer's specifications at the Bioscience Core Lab at KAUST, Saudi Arabia.

### 2.8 Identification of Symbiodiniaceae taxa

Sequencing data were analyzed with the SymPortal engine (Hume et al., 2019), a platform for phylogenetically resolving Symbiodiniaceae taxa using ITS2 amplicon data. SymPortal was run locally and all samples, including samples from the isolate symbiont cultures used to perform the inoculations, were analyzed together. Briefly, this software identifies sets of specific ITS2 sequences that reoccur in sufficient numbers of samples and considers them as “defining intra‐genomic variants” which in turn are then used to characterize an “ITS2 type profile” representative of putative Symbiodiniaceae taxa. Different terms have been used over time, often in an interchangeable manner, to describe taxonomic units of resolution within this group (e.g., “ITS2 type,” “ITS2 profile,” “type,” “subtype,” “clade,” “subclade”). Thus, for the purposes of this study we restricted our use to “majority ITS2 sequence” and “ITS2 type profile.” As defined by SymPortal, “majority ITS2 sequence”’ refers to the most abundant sequence(s) in each of the samples that have a “type profile,” which defines a putative taxon. For example, *S. linucheae* was characterized by the specific A4‐A4m ITS2 type profile, with A4 as the most abundant ITS2 sequence. Additional details on SymPortal are provided in Methods S1.

### 2.9 Statistical analyses

The response variables were analyzed using both analyses of variance (ANOVAs) and linear mixed models (LMMs). All data were first checked for normality and homoscedasticity using standardized residual plots and Q–Q plots and, if required, ln or ln (*x* + 1) transformations were applied. Symbiont cell densities were analyzed with a one‐way ANOVA using host‐symbiont combination as a fixed explanatory variable in R version 3.5.1 (R Core Team, 2018). If significant terms were detected, Tukey pairwise comparisons were conducted post hoc to determine where significant differences occurred.

Gross photosynthesis and respiration (pmol C cell^−1^ hr^−1^), and P:R ratios were analyzed using repeated‐measures LMMs in SPSS (Released 2013) as described previously (Klein et al., 2017). For each dependent variable, the fixed factors were host genotype, symbiont type (based on the majority ITS2 sequence), and temperature, which was the repeated measure. In all cases, several repeated covariance types (e.g., AR(1), AR(1) heterogeneous, CS) were investigated to assess the model‐of‐best fit by comparing numerous goodness‐of‐fit statistics (e.g., −2 restricted log likelihood, Akaike's information criterion and Bayesian information criterion). Preliminary analyses of the response variables included random factors (or blocks) to test for potential bias associated with the oxygen sensor ID fitted to each individual chamber, water tub ID in which chambers were submerged, and day on which the assay was performed. We used estimates of covariance parameters and the Wald *Z* test of simultaneous coefficients to assess the potential redundancy of these terms in each analysis. If any of these were revealed to significantly affect the fit of the model, they were retained to account for associated variance but were otherwise removed and the analyses rerun. Photochemical efficiency values (*Fv/Fm*) for each host–symbiont combination subjected to long‐term heat stress were analyzed using LMMs with day as the repeated measure. Likewise, AEs for each response variable measured were also analyzed with LMMs but without repeated covariance structure. For all LMM analyses, estimated marginal means (least‐squares means) were used to determine which means differed for the significant, highest‐order terms.

## 3 RESULTS

### 3.1 Colonization success

We identified three main putative symbiont taxa based on the majority ITS2 sequence: B1, A4, and A1 corresponding to *B. minutum* (SSB01), *S. linucheae* (SSA01), and *S. microadriaticum* (RS‐A), respectively (Table 1). The B1 taxon was particularly interesting as it exhibited distinct type profiles (i.e., different genotype representatives) for the Hawaii and Red Sea strains, which were designated as B1 and RS‐B1, respectively (Figure S1). In some cases, the presence of both A4 and A1 taxa also resulted in the recovery of the artefactual A4/A1 genotype (see Methods S1). Sequences belonging to other taxa (i.e., *Cladocopium* and *Durusdinium* sp.) with relative abundances below 1%, on the other hand, were classified as “others.”

Certainly, functional significance of rare and low abundant background Symbiodiniaceae taxa cannot be underestimated (Ziegler, Eguíluz, Duarte, & Voolstra, 2018). However, for the purpose of this study, we only considered individuals with majority ITS2 sequences corresponding to at least 80% of the targeted symbiont taxon. Thus, based on this, colonization success (i.e., proportion of individuals colonized by the desired symbiont) of each host–symbiont combination was examined and, on a case‐by‐case basis individuals were grouped according to the main symbiont they harbored (Table S1). It is noteworthy that the original isolate used to perform Red Sea inoculations was ~70% type A1 (Figure S1), yet the symbiont composition of homologous RS‐Red Sea Aiptasia was mainly characterized by the A4/A1 type (Figure S2; Discussion S1). Even though we only tested Symbiodiniaceae that are native to Aiptasia, we still observed limited interpartner compatibility in some cases. Specifically, holobionts with heterologous Red Sea taxa did not maintain a stable symbiosis (i.e., they died or reverted to their original symbiont composition) beyond 1 year after initial inoculations (Table S1; Figure S3).

### 3.2 Photosynthesis and respiration

**FIGURE 2:**
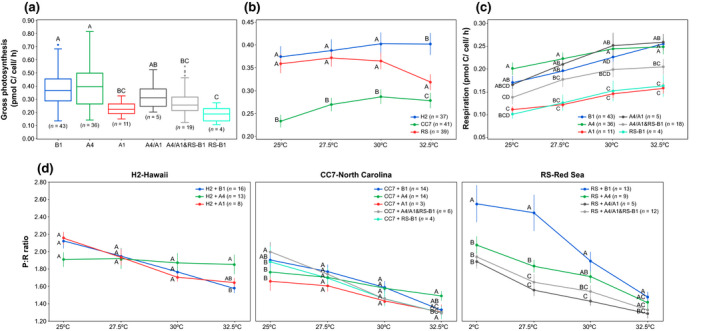
(a) Symbiont identity significantly affected gross photosynthesis rates yet (b) host genotype moderated the response to temperature. (c) Respiration rates varied with symbiont identity in response to temperature. (d) Photosynthesis to respiration (P:R) ratios across temperature increments for H2‐Hawaii, CC7‐North Carolina, and RS‐Red Sea Aiptasia harboring different Symbiodiniaceae taxa. All response variables are shown as mean values ±1 *SE*. Pairwise comparisons were carried out within each temperature increment. Letters next to data points indicate similarities (e.g., AA) or differences (e.g., AB) between host and/or symbiont types, as determined by estimated marginal means. Sample size (*n*) for each case is indicated in the legend

Thermal performance varied greatly among host–symbiont combinations (Table S2). Overall, rates of gross photosynthesis differed among symbiont types, resulting in a significant main effect. However, the response of gross photosynthesis to thermal stress depended only on host identity, resulting in a significant host × temperature interaction. Surprisingly, rates of gross photosynthesis did not significantly vary between B1 and A4 symbionts but showed differences within *Symbiodinium* taxa (A4 and A1), and intraspecifically between B1 and RS‐B1 (Figure 2a). Overall, rates of productivity in the H2‐Hawaii holobionts were consistently higher across all temperatures, regardless of symbiont composition (Figure 2b). Respiration, on the other hand, depended on symbiont type, thus resulting in a significant symbiont × temperature interaction (Figure 2c). Interestingly, RS‐Red Sea strains exhibited lower respiration compared to others, yet, when in heterologous combinations (i.e., H2‐Hawaii and CC7‐North Carolina hosts harboring RS taxa), these had the highest respiration rates (Figure S6a). Increased respiratory rates also corresponded to the highest cell densities (Figure S5) which in turn explained the high productivity (Figure S6b; see Discussion S3). Photosynthesis to respiration ratio (P:R) responses to temperature differed according to host genotype and depended upon symbiont identity, resulting in a significant three‐way host × symbiont × temperature interaction, further highlighting the interplay between both partners (see below). Respiration rates increased much faster than photosynthesis with warming, leading to declining P:R ratios (Figure 2d) over time with accumulated stress. In agreement with our observations of gross photosynthesis, H2‐Hawaii Aiptasia also displayed the highest P:R ratios under the highest temperature of 32.5°C (Figure 2d).

### 3.3 Photochemical efficiency (*Fv*/*Fm*)

**FIGURE 3:**
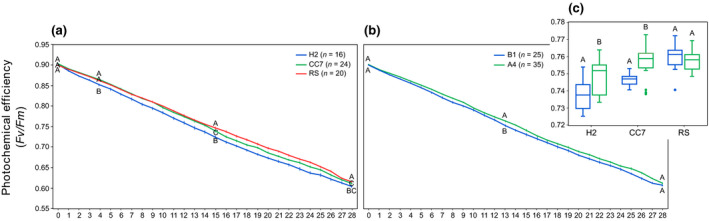
Mean (±1 *SE*) photochemical efficiencies of different (a) host genotypes, (b) symbiont types, and (c) host–symbiont combination. Letters indicate similarities (e.g., AA) or differences (e.g., AB), as determined by estimated marginal means. Sample sizes (*n*) for host genotypes (a) and symbiont types (b) are indicated in the legends

Here, we characterized the thermal acclimation potential of the various host–symbiont combinations (noteworthy Red Sea hosts nor symbionts could be tested in this experiment; see Table S1) during long‐term heat stress by monitoring daily changes in symbiont photochemical efficiency (*Fv*/*Fm*). After 28 days at 32°C, *Fv*/*Fm* dropped from ~0.9 (on day 0 at 25°C) to ~0.6. Symbionts’ *Fv*/*Fm* depended on host and time (Table S3). CC7‐North Carolina and RS‐Red Sea genotypes exhibited higher heat tolerance than H2‐Hawaii (Figure 3a) as was also the case for A4 taxa compared to B1 (Figure 3b). Overall differences between the various host–symbiont combinations were also detected (Figure 3c), with those harboring B1 displaying lower yields. Although a significant host × symbiont × day interaction was not detected, we could nonetheless observe variability among host–symbiont combinations over time (Figure S7). We observed lower *Fv*/*Fm* for H2‐Hawaii and CC7‐North Carolina with B1 compared to the same genotypes harboring A4 taxa, which was further associated with a greater loss of symbionts (Figure S8).

### 3.4 Thermal sensitivity of metabolic rates

**FIGURE 4:**
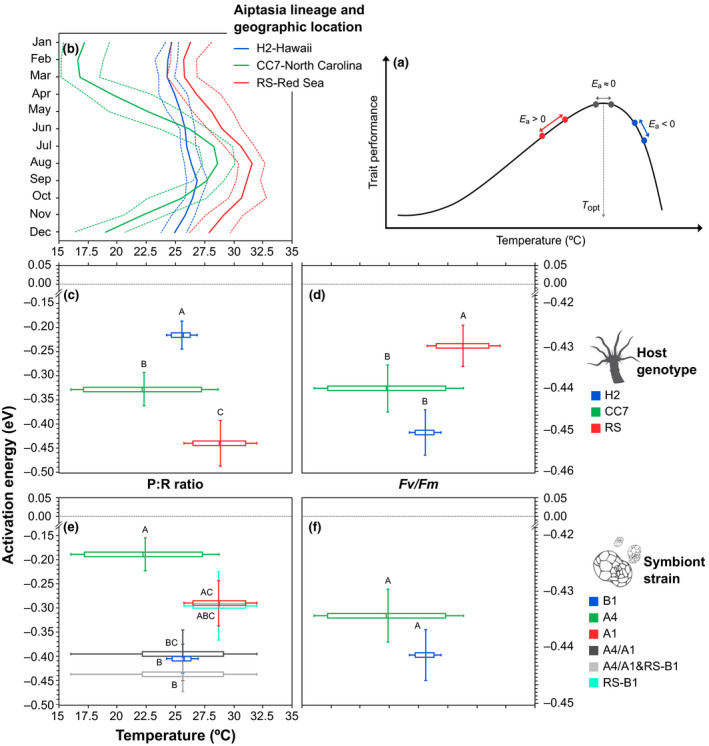
(a) Thermal response of physiological and metabolic processes has at least two different regimes: one above their optimal performance temperature (*T*_opt_), rise, and one below their optimum, fall. The Boltzmann–Arrhenius model involves calculating activation energies (*E*_a_) from the temperature dependence of reaction rates. Here *E*_a_ < 0 (i.e., negative values) indicate a decrease in the trait response with increasing temperature, *E*_a_ ≈ 0 indicate no change in the trait response with increasing temperature and *E*_a_ > 0 (i.e., positive values) indicate an increase in the trait response with warming. The range of the rise regime is where organisms normally operate (highlighted by the green area), whereas the fall component (blue) is usually steeper and typically indicates biological collapse. (b) Thermal regimes of geographical locations of origin for the three Aiptasia lineages used here: Hawaii (Kāne'ohe Bay), North Carolina (Wilmington), and Red Sea (Al Lith, Saudi Arabia). Solid line denotes average, and dashed lines represent maximum and minimum sea surface temperatures (data taken from www.seatemperature.org; figure modified from Cziesielski et al., 2018). Box and whisker plots show the median and range of temperature variability where host (c, d) and symbiont taxa (e, f) originate versus the mean (±1 *SE*) activation energy (eV) associated to P:R ratios and photochemical efficiency (*Fv/Fm*). Thermal variation for symbiont taxa containing the artefactual “A4/A1” genotype was calculated based on the two corresponding locations (North Carolina and Red Sea). Letters next to data points indicate similarities (e.g., AA) or differences (e.g., AB) as determined by estimated marginal means

We characterized the thermal dependence of metabolic processes from the associated activation energy (*E*_a_), calculated following the Boltzmann–Arrhenius model (Brown, Gillooly, Allen, Savage, & West, 2004; Dell et al., 2011); which is mathematically equivalent to an effect size per unit warming (Marbà et al., 2015), thereby facilitating comparisons of the magnitude of response across biological traits (Figure S9). *E*_a_ estimates reported here were calculated in a manner where a value of zero indicates no (or limited) thermal dependence of the response, whereas greater deviations from zero (positive or negative) dictate increasing thermal dependence (Brown et al., 2004; Dell et al., 2011; see Figure 4a). Models comparing *E*_a_ of photosynthesis, respiration, and P:R ratios revealed significant host × symbiont interactions in all cases (see Table S4). Notably, H2‐Hawaii holobionts differed from the rest as they exhibited smaller *E*_a_ (Figure S9a). *E*_a_ associated with photoinhibition of PS II depended on host genotype and not symbiont taxa (Table S4; Figure S9b), and followed the order H2‐Hawaii > CC7‐North Carolina > RS‐Red Sea. Overall, the magnitude of the response to temperature of the holobiont appeared to be strongly related with its location of origin. As H2‐Hawaii originates from a lower temperature and less variable environment, mean *E*_a_ for P:R ratios was much lower relative to CC7‐North Carolina and RS‐Red Sea Aiptasia, where local environment is warmer and more variable (Figure 4b,c). In turn, this also corresponded with the degree of deterioration (*Fv*/*Fm*) suffered (Figure 4d). Surprisingly, we observed the opposite pattern for symbionts (Figure 4e). A4 taxa (*S. linucheae*, homologous to CC7‐North Carolina) seemed to be more affected by temperature than B1 from Hawaii despite originating from significantly more fluctuating thermal settings. Heat sensitivity (i.e., *Fv*/*Fm*) of B1 was not different from A4 either (Figure 4f).

## 4 DISCUSSION

### 4.1 Symbiosis specificity in a cnidarian holobiont

Even though all symbiont strains initially colonized Aiptasia, long‐term interpartner compatibility was still limited in some cases (see Discussion S2), especially for hosts harboring heterologous RS taxa. Death of the H2‐Hawaii + RS combination, for example, was not surprising as other studies (Starzak et al., 2014; Tortorelli, Belderok, Davy, McFadden, & van Oppen, 2020) have already shown high mortality due to colonization of incompatible symbionts. For CC7‐North Carolina + RS, it is possible that if anemones were not completely bleached before inoculating them, communities could have shuffled to favor the homologous symbiont. It is important to take the latter into account as here we detected the presence of symbiont types (i.e., “others”) that were not included in the inoculation mix. Some individuals might not have been fully aposymbiotic at the time we performed the inoculations, thus contributing to the proliferation of “unwanted” symbionts. Similarly, we cannot exclude potential contamination while maintaining the animals, feeding (i.e., food was taken from a common batch) and/or subsequently during PCR amplification.

### 4.2 Photophysiological performance is driven by partner specificity

Consistent with previous reports showing the impacts of symbiont diversity on the physiology (Gabay et al., 2018; Goulet et al., 2005; Hawkins, Hagemeyer, Hoadley, Marsh, & Warner, 2016; Hoadley et al., 2019; Rädecker et al., 2018; Starzak et al., 2014), metabolic fluxes (Matthews et al., 2017, 2018), and protein expression levels (Medrano, Merselis, Bellantuono, & Rodriguez‐Lanetty, 2019; Sproles et al., 2019), we also observed differences in thermal performance among host–symbiont combinations. Not only are metabolic capabilities of Symbiodiniaceae are highly variable, but host identity can also play a fundamental role in determining the physiological response of the symbiont. For example, *B. minutum* is known to be thermally sensitive (Cziesielski et al., 2018; Grégoire, Schmacka, Coffroth, & Karsten, 2017; Rädecker et al., 2018; Robison & Warner, 2006; Swain, Chandler, Backman, & Marcelino, 2017) yet a more beneficial symbiont, at least in Aiptasia (Gabay et al., 2018; Rädecker et al., 2018; Starzak et al., 2014). Taxa from the genus *Symbiodinium*, on the other hand, can fix carbon at higher rates than other symbionts but tend to translocate less photosynthates to the host (Rädecker et al., 2018). Furthermore, the thermal response of *Durusdinium trenchii* can vary greatly depending on the coral species (Hoadley et al., 2019; Rädecker et al., 2018). Accordingly, our study demonstrates that different symbiont strains have a substantial impact on the stability and functionality of the symbiosis but that it is the host that largely determines holobiont productivity in response to heat stress.

Noteworthy, we also found that RS‐B1 (i.e., specific ITS2 type profiles found only in RS‐Red Sea Aiptasia) is distinctly different from B1 from Hawaii; and while questions remain regarding possible genotypic differences between these two, our data suggest that RS‐B1 might be indeed a different species or at least a different “eco‐type” worthy of further study. These observations highlight the importance of considering fine‐scale differences when comparing performance of different symbiont strains, as Symbiodiniaceae is already known to be greatly diverse at inter‐ and intraspecific levels (LaJeunesse et al., 2018).

### 4.3 Extent of thermal resilience depends on host–symbiont partnership

Most studies have suggested that it is the genetic identity and eco‐physiological attributes of Symbiodiniaceae that ultimately determine thermal acclimation of corals (Berkelmans & van Oppen, 2006; Cziesielski et al., 2018; Howells et al., 2011; Sampayo, Ridgway, Bongaerts, & Hoegh‐Guldberg, 2008). This is despite the increasing evidence that the host also exhibits signs of stress and may even be impaired before the symbiont (reviewed in Oakley & Davy, 2018). For example, it has recently been shown that host‐derived production of reactive oxygen species increases days before detecting bleaching or photoinhibition (Krueger et al., 2015; Oakley et al., 2017). Thus, even with the symbiont's own coping mechanisms, its performance still relies on (phenotypic and genotypic) plasticity of the host to respond under stress (Bellantuono, Hoegh‐Guldberg, & Rodriguez‐Lanetty, 2012; Kenkel & Matz, 2016). Certainly, the ability of the host to adapt to alternative environments plays a strong role (perhaps more than what has been acknowledged) in predicting thermal tolerance (Hoadley et al., 2019; Howells, Abrego, Meyer, Kirk, & Burt, 2016; Kenkel & Matz, 2016; Morikawa & Palumbi, 2019; Palumbi et al., 2014).

Here, and in line with Hoadley et al. (2019), we show that even within heat tolerant taxa like *S. microadriaticum* (Cziesielski et al., 2018; Díaz‐Almeyda et al., 2017; Swain et al., 2017; and potentially *Breviolum* types isolated from the RS lineage), host‐dependent (physiological) differences can strongly affect the overall thermal sensitivity of the holobiont. We see that even if the thermal response (measured as *Fv*/*Fm*) of H2‐Hawaii is improved when harboring heterologous, compatible symbionts (A4), it is still below CC7‐North Carolina and RS‐Red Sea holobionts. Indeed, strain‐specific responses have been identified for Aiptasia; particularly, RS‐Red Sea stands out as a more heat tolerant genotype (Cziesielski et al., 2018).

### 4.4 Local adaptation accounts for species‐specific responses to thermal stress

One species can be composed of different, locally adapted populations that differ in their ability to physiologically respond to changes in their environment (Bennett, Duarte, Marbà, & Wernberg, 2019), such that adaptation to a specific set of conditions can constrain and/or cause distribution shifts (Valladares et al., 2014). Populations are expected to adapt so that local genotypes have higher fitness in their native habitat than those from more distant populations (Kawecki & Ebert, 2004; Sanford & Kelly, 2010). Thus, local adaptation not only determines the spatial and temporal patterns of distribution, abundance, and ecological niches of populations of a given species (Valladares et al., 2014) but might well predict its response to environmental disturbances (Bennett et al., 2019; Kawecki & Ebert, 2004; Sanford & Kelly, 2010).

We tested the effect of temperature on the physiological performance of various Aiptasia lineages (host and symbionts) that originate from different geographic regions, and consequently have adapted to distinct thermal regimes. The locations of origin of the Aiptasia lineages tested exhibit different thermal regimes (Figure 4b), North Carolina has large seasonal fluctuations (almost 15°C), whereas temperatures in Hawaii are comparatively stable (temperature range <5°C). Organisms exhibit different levels of physiological compensation (i.e., phenotypic plasticity in the expression of metabolism) when coping with alternative conditions so that individuals from more variable environments show higher plasticity that might increase their ability to respond to extremes (Chevin & Hoffmann, 2017). For instance, a recent global analysis indicated a potentially fundamental role of high frequency temperature variability in reducing the severity of coral bleaching (Safaie et al., 2018). Thus, adding to the body of work shows that previous exposure to a thermally variable environment contributes substantially to the holobiont thermal tolerance beyond that provided by heat‐resistant symbionts alone (Carilli, Donner, & Hartmann, 2012; Howells, Berkelmans, van Oppen, Willis, & Bay, 2013; Oliver & Palumbi, 2011).

We hypothesized that temperature dependence of metabolic rates, characterized here as P:R ratio, reflects plasticity in response to heat stress. We therefore expected that higher plasticity would result in increased holobiont thermotolerance. Our data suggest that hosts from locations with lower and less variable temperatures show significantly lower plasticity (i.e., the ability to adjust their physiology in response to warming); that is, H2‐Hawaii is less plastic than CC7‐North Carolina and RS‐Red Sea Aiptasia. Indeed, H2‐Hawaii was the most susceptible to thermal stress (as reflected by lower photochemical yields and greater loss of symbionts) compared to the others. Furthermore, differences in the plastic responses vary among host and symbiont partners but specifically, that the former determines, in great part, acclimation to stress (as it has higher physiological plasticity). One possible explanation could be the dependence of the symbiont on the host (i.e., the symbiont relies on the host to provide a suitable environment that supports its functioning). Indeed, it has been shown that *in hospite* nutrient availability for the symbiont differs depending on the associated host (Rädecker et al., 2018), so that performance of the symbiont (e.g., gross productivity and carbon translocation) may be largely attributed to variations in host metabolism. Moreover, our results point toward a remarkable effect of local adaptation on holobiont metabolic plasticity.

## 5 CONCLUSIONS

This study provides valuable insight into the mechanisms (phenotypic plasticity and local adaptation) underlying the heat stress response of a cnidarian model system and highlights the role of the host and its natural local environment in this process. Coles and Jokiel (1977) first showed differences in the photosynthetic and respiratory capacities of corals in relation to their habitat and thermal histories. Here we build upon this knowledge, and further demonstrate that acclimation mechanisms are consistent with local adaptation to specific conditions; particularly, that individuals from a more thermally variable environment have the ability to dynamically regulate their response to temperature stress. We show that local adaptation may be a strong determinant of symbiosis specificity and as such, inoculation with more heat tolerant partners, even if naturally found in Aiptasia, does not automatically improve thermal resilience of the holobiont.

As global warming intensifies, novel communities and ecological networks are expected to emerge through species turnover and shifts in their distribution (Lurgi, López, & Montoya, 2012; Valladares et al., 2014) via phenotypic plasticity and/or rapid evolution (Hoffmann & Sgrò, 2011; Reusch, 2014; Torda et al., 2017). Thus, understanding the evolutionary constraints and trade‐offs of symbiosis is necessary to develop more realistic models of species survival (of symbiotic cnidarians) and its ecological consequences for future oceans. Particularly, quantifying the amount of plasticity and adaptive potential in metabolic rates may be important to forecast how organisms will cope with, in this case, warmer oceans (Bennett et al., 2019).

We are, nevertheless, cautious in drawing conclusions about thermal adaptation of symbiotic cnidarians, given the potentially oversimplified nature of our experimental approach. Conditions in laboratory settings are far more simplistic than the complex heterogeneity innate to natural environments, and so rates of adaptation here might not be consistent with those in nature where other important environmental traits vary with warming. Furthermore, all host and symbiont strains we tested here are not wild populations but have been cultured under the same, stable thermal regime for a considerable time (at least 2 years). Therefore, we cannot exclude a certain degree of acclimation or even adaptation to the culture conditions, which could account for some of the weak effects we observed here; especially with regard to the symbionts, which have a much shorter life cycle and, hence, higher evolutionary rates than the host (Chakravarti et al., 2017; Chakravarti & van Oppen, 2018; Pandolfi, Connolly, Marshall, & Cohen, 2011). More importantly, it should be noted that only one biological replicate (i.e., genotype) was examined for each geographic location.

Our results indicate that local adaptation is a holobiont trait (defined by the host and the symbionts alike) that cannot be retained when symbionts are transferred to new and unfamiliar hosts. These findings suggest that we may be limited in our capacity to manipulate cnidarian symbioses in light of climate change and that experimental acclimation (i.e., hardening) of the different, coevolved partners (Buerger et al., 2020; Chakravarti et al., 2017; Chakravarti & van Oppen, 2018; Cziesielski, Schmidt‐Roach, & Aranda, 2019) may be a more promising method to increase the persistence of corals in the Anthropocene.

## Supporting information

Supplementary Material

## Data Availability

Sequencing data are available at NCBI under project number PRJNA577376.
