# Supplementary material for: Unfamiliar partnerships limit cnidarian holobiont acclimation to warming
Source: Glob Chang Biol. 2020 Jul 26;26(10):5539–53. doi: 10.1111/gcb.15263 (PMC7539969; doi:10.1111/gcb.15263)
Supplement: Supplementary file 1 — Supplementary Material [file GCB-26-5539-s001.docx]

**SUPPORTING INFORMATION**

**Methods S1. Considerations on SymPortal**

Although the ITS2 marker has been widely used to genetically characterize Symbiodiniaceae taxa, the multicopy nature of this gene complicates its use. A single cell can contain hundreds to thousands of rRNA gene copies; thus, variations among copies can give rise to an extraordinarily high intragenomic ITS2 diversity. SymPortal (Hume et al., 2019) makes use of this intragenomic diversity (which it refers to defining intragenomic variants or DIVs) to identify genotype representatives (type profiles) of putative Symbiodiniaceae taxa. However, despite being capable of resolving genetic delineations using the ITS2 marker at a finer scale than before (at least without the use of additional markers), SymPortal’s approach has some inherent limitations (see Hume et al., 2019). Particularly, if Symbiodiniaceae taxa co-occur in a sufficient number of samples (SymPortal currently uses an arbitrary support threshold of 4, Ben Hume *personal communication*), the ITS2 sequences returned from several taxa may be reported as being representative of a single taxon (i.e. super type). Though this is not a concern for corals, as they usually harbor only one dominant symbiont species from a given genus, it seems to be a problem for our Aiptasia samples (i.e. artificially created organisms). Indeed, preliminary analyses revealed the artifactual genotype A1/A4 (i.e. presence of both A1 and A4 taxa).

**Discussion S1. Changes in the symbiont composition of Red Sea Aiptasia**

We observed a significant change in the symbiont composition between RS-Red Sea anemones that were collected in the wild (March 2015) and individuals that had been kept in the laboratory for at least three years (sampled on August 2018) (Fig. S4). These differences were mainly attributed to the presence of A4 symbionts (as shown by the A4/A1 genotype) and disappearance of *Cladocopium* and *Durusdinium* taxa (here denoted as ‘others’). Very often, the microbial composition of laboratory-maintained organisms is different to their wild counterparts (Gaikwad et al., 2016) as a result of the distinct conditions of controlled settings in the laboratory versus a dynamic environment in the wild. Therefore, considering the average temperature profiles of the locations from which Aiptasia were originally obtained (Cziesielski et al., 2018), it is possible that individuals reared long-term at 25 °C could have acclimated to winter conditions. Even the lowest temperatures in the central Red Sea during the winter season can be above 25 °C. Indeed, it has been shown that corals can experience quiescence (i.e. dormancy) during cold temperatures, a phenomenon that can drive significant changes in the microbial communities (Grace, 2017; Sharp et al., 2017). We can only speculate, however, as the biology of quiescence still remains understudied.

**Discussion S2. Symbiosis specificity in Aiptasia**

Aiptasia has made possible to study the flexibility of this cnidarian-algal symbiosis by performing experimental inoculations with heterologous symbionts. Yet, studies to-date have only investigated the dynamics of short-term (12 weeks maximum) inoculations (Belda-Baillie et al., 2002; Gabay et al., 2018; Starzak et al., 2014). Here, we studied heterologous symbioses that remained stable for at least six months and longer (> 1 year). Despite only testing a limited number (*n* = 3) of taxa, however, the strains we used in this study naturally occur as main symbionts in the different Aiptasia lineages.

Differential success of heterologous symbionts in Aiptasia has already been observed. Failure of certain taxa such as the free-living *Effrenium voratum* (referred as SSE02) to establish symbiosis with both larval and adult Aiptasia (or acroporid corals) has been reported on several occasions (Gabay et al., 2018; Hambleton et al., 2014; Wolfowicz et al., 2016). Compatibility can also vary within the same genus; both SSA01 and SSA02 strains (*Symbiodinium*) can efficiently infect different hosts while SSA03 cannot be found in abundances of more than 1-2 algal cells per larvae (Hambleton et al., 2014; Wolfowicz et al., 2016), for example. Likewise, taxa from the genus *Cladocopium* seem incapable of proliferating in Aiptasia even after three months of being taken up (Belda-Baillie et al., 2002; Gabay et al., 2018). Further, evidence has shown that non-native taxa, if accepted, reach much higher cell densities than homologous symbionts (Hambleton et al., 2014; Rädecker et al., 2018; Starzak et al., 2014). Our data showed similar symbiont densities cross all naturally occurring host-symbiont combinations with 1.43 x 10^7^ (± 6.58 x 10^5^ SE) cells/mg protein. This density was not significantly different from that attained by experimental subjects, with the exception of H2-Hawaii and CC7-North Carolina anemones harboring A1 and A4/A1&RS-B1 and RS-B1 taxa, respectively (Fig. S5).

As the host must regulate the symbiont population for a stable symbiosis to persist (Cunning & Baker, 2014), it is possible that certain mechanisms are somehow hindered in heterologous symbiosis. Hence, it is not surprising that even if colonization was initially successful for H2-Hawaii + A1, all the remaining individuals of this host-symbiont combination died shortly after concluding the respirometry assays (~ 6 months after inoculations). CC7-North Carolina anemones infected with RS-Red Sea taxa did not sustain long-term symbiosis either. Instead, most of the individuals reverted to their homologous A4 symbiont composition (Fig. S2). Noteworthy symbiont densities shown here are of the same order of magnitude as previously reported for Aiptasia in other studies (Gabay et al., 2018; Gegner et al., 2017; Hawkins et al., 2016).

**Discussion S3. Functional performance depends on symbiont abundance**

Symbiont abundance is not only and indicator of bleaching but also a critical determinant of the state of symbiosis. In a density-dependent framework, symbiont abundance has a fundamental role in the dynamic interactions between the holobiont and its abiotic environment, which ultimately defines the performance of the symbiosis (reviewed in Cunning & Baker, 2014). Essentially, the photosynthetic capacity of symbionts depends on the light fields they experience. When are low, each cell receives more light but as their abundance increases, self-shading reduces the amount of incident light available they can capture. Thus, leading to an intense competition for limited resources that decreases the gross benefits for the host (i.e. carbon fixation) while, at the same time, increasing the energetic costs of maintaining high symbiont densities (e.g. removal of oxygen radicals, damage repair from photo-oxidative stress). Since symbionts contribute to the total holobiont respiration, respiratory rates are also expected to increase linearly with symbiont densities (Hawkins et al., 2016; Hoogenboom et al., 2010) so that the carbon contribution of each individual symbiont is reduced or even cancelled, making the host more susceptible to bleaching (Cunning & Baker, 2014). Hence, in order for the host to build and maintain energy reserves (i.e. P:R ratio > 1), an ‘optimum’ symbiont density must exist (Hoogenboom et al., 2010).

**REFERENCES**

Belda-Baillie, C. A., Baillie, B. K., & Maruyama, T. (2002). Specificity of a Model Cnidarian-Dinoflagellate Symbiosis. *The Biological Bulletin*, *202*(1), 74–85. https://doi.org/10.2307/1543224

Cunning, R., & Baker, A. C. (2014). Not just who, but how many: the importance of partner abundance in reef coral symbioses. *Frontiers in Microbiology*, *5*, 400. https://doi.org/10.3389/fmicb.2014.00400

Cziesielski, M. J., Liew, Y. J., Cui, G., Schmidt-Roach, S., Campana, S., Marondedze, C., & Aranda, M. (2018). Multi-omics analysis of thermal stress response in a zooxanthellate cnidarian reveals the importance of associating with thermotolerant symbionts. *Proceedings. Biological Sciences*, *285*(1877), 20172654. PubMed. https://doi.org/10.1098/rspb.2017.2654

Gabay, Y., Weis, V. M., & Davy, S. K. (2018). Symbiont Identity Influences Patterns of Symbiosis Establishment, Host Growth, and Asexual Reproduction in a Model Cnidarian-Dinoflagellate Symbiosis. *The Biological Bulletin*, *234*(1), 1–10. https://doi.org/10.1086/696365

Gaikwad, S. S., Chowdhury, S. P., Shouche, Y. S., Ghaskadbi, S., & Ghaskadbi, S. (2016). Laboratory maintained and wild populations of Hydra differ in their microbiota. *Annals of Microbiology*, *66*(2), 931–935. https://doi.org/10.1007/s13213-015-1177-z

Gegner, H. M., Ziegler, M., Rädecker, N., Buitrago-López, C., Aranda, M., & Voolstra, C. R. (2017). High salinity conveys thermotolerance in the coral model Aiptasia. *Biology Open*, *6*(12), 1943. https://doi.org/10.1242/bio.028878

Grace, S. (2017). Winter Quiescence, Growth Rate, and the Release from Competition in the Temperate Scleractinian Coral Astrangia poculata (Ellis & Solander 1786). *Northeastern Naturalist*, *24*(sp7). https://doi.org/10.1656/045.024.s715

Hambleton, E. A., Guse, A., & Pringle, J. R. (2014). Similar specificities of symbiont uptake by adults and larvae in an anemone model system for coral biology. *The Journal of Experimental Biology*, *217*(9), 1613. https://doi.org/10.1242/jeb.095679

Hawkins, T. D., Hagemeyer, J. C. G., Hoadley, K. D., Marsh, A. G., & Warner, M. E. (2016). Partitioning of Respiration in an Animal-Algal Symbiosis: Implications for Different Aerobic Capacity between Symbiodinium spp. *Frontiers in Physiology*, *7*, 128–128. PubMed. https://doi.org/10.3389/fphys.2016.00128

Hoogenboom, M., Beraud, E., & Ferrier-Pagès, C. (2010). Relationship between symbiont density and photosynthetic carbon acquisition in the temperate coral Cladocora caespitosa. *Coral Reefs*, *29*(1), 21–29.

Hume, B. C. C., Smith, E. G., Ziegler, M., Warrington, H. J. M., Burt, J. A., LaJeunesse, T. C., Wiedenmann, J., & Voolstra, C. R. (2019). SymPortal: A novel analytical framework and platform for coral algal symbiont next-generation sequencing ITS2 profiling. *Molecular Ecology Resources*, *19*(4), 1063–1080. https://doi.org/10.1111/1755-0998.13004

Rädecker, N., Raina, J.-B., Pernice, M., Perna, G., Guagliardo, P., Kilburn, M. R., Aranda, M., & Voolstra, C. R. (2018). Using Aiptasia as a Model to Study Metabolic Interactions in Cnidarian-Symbiodinium Symbioses. *Frontiers in Physiology*, *9*, 214–214. PubMed. https://doi.org/10.3389/fphys.2018.00214

Sharp, K. H., Pratte, Z. A., Kerwin, A. H., Rotjan, R. D., & Stewart, F. J. (2017). Season, but not symbiont state, drives microbiome structure in the temperate coral Astrangia poculata. *Microbiome*, *5*(1), 120. https://doi.org/10.1186/s40168-017-0329-8

Starzak, D. E., Quinnell, R. G., Nitschke, M. R., & Davy, S. K. (2014). The influence of symbiont type on photosynthetic carbon flux in a model cnidarian–dinoflagellate symbiosis. *Marine Biology*, *161*(3), 711–724. https://doi.org/10.1007/s00227-013-2372-8

Wolfowicz, I., Baumgarten, S., Voss, P. A., Hambleton, E. A., Voolstra, C. R., Hatta, M., & Guse, A. (2016). Aiptasia sp. larvae as a model to reveal mechanisms of symbiont selection in cnidarians. *Scientific Reports*, *6*, 32366.


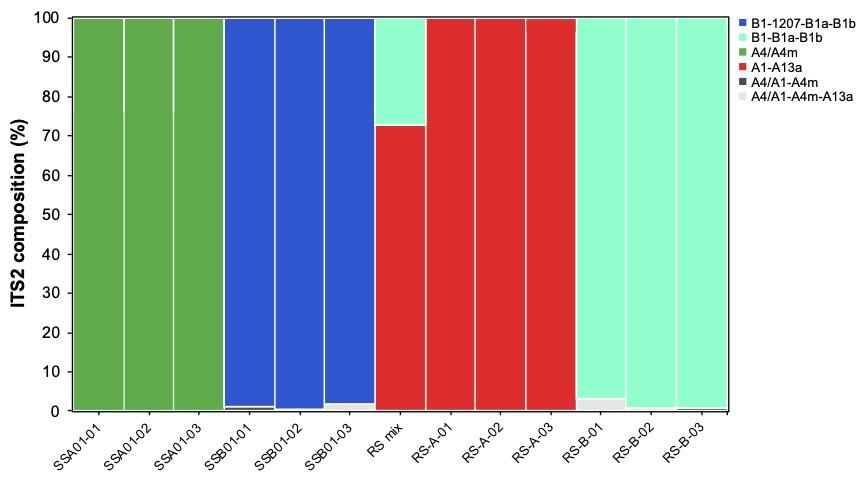


**Figure S1.** ITS2 type profiles of the Symbiodiniaceae cultures used to perform inoculations: SSA01, SSB01 and, Red Sea clade A and B strains isolated from a Red Sea Aiptasia (RS mix). Each symbiont strain in this study is represented by only one culture line from which three technical replicates were independently extracted.

**Table S1.** Detailed overview of all experimental Aiptasia-Symbiodiniaceae combinations tested in this study. Putative taxa are based on the majority ITS2 sequence as determined by SymPortal. Homologous relationships are highlighted in bold. Sample size is indicated in each case.

|  | *host* | | |
| --- | --- | --- | --- |
| *symbiont* | H2-Hawaii | CC7-North Carolina | RS-Red Sea |
| SSA01 | H2 + A4 (*n* = 13)^a^ | **CC7 + A4 (*n* = 14)**^a^ | RS + A4 (*n* = 9)^a^ |
|  | H2 + A4 (*n* = 8)^b^ | **CC7 + A4 (*n* = 17)**^b^ | RS + A4 (*n* = 10)^b^ |
| SSB01 | **H2 + B1 (*n* = 16**)^a^ | CC7 + B1 (*n* = 14)^a^ | RS + B1 (*n* = 13)^a^ |
|  | **H2 + B1 (*n* = 8)**^b^ | CC7 + B1 (*n* = 7)^b^ | RS + B1 (*n* = 10)^b^ |
| RS | H2 + A1 (*n* = 8)^a^ | CC7 + A1 (*n* = 3)^a^  CC7 + A4/A1&RS-B1 (*n* = 6)^a^  CC7 + RS-B1 (*n* = 4)^a^ | **RS + A4/A1 (*n* = 5)**^a^  **RS + A4/A1&RS-B1 (*n* = 12)**^a^ |

^a^ acute heat stress experiment

^b^ long-term heat exposure


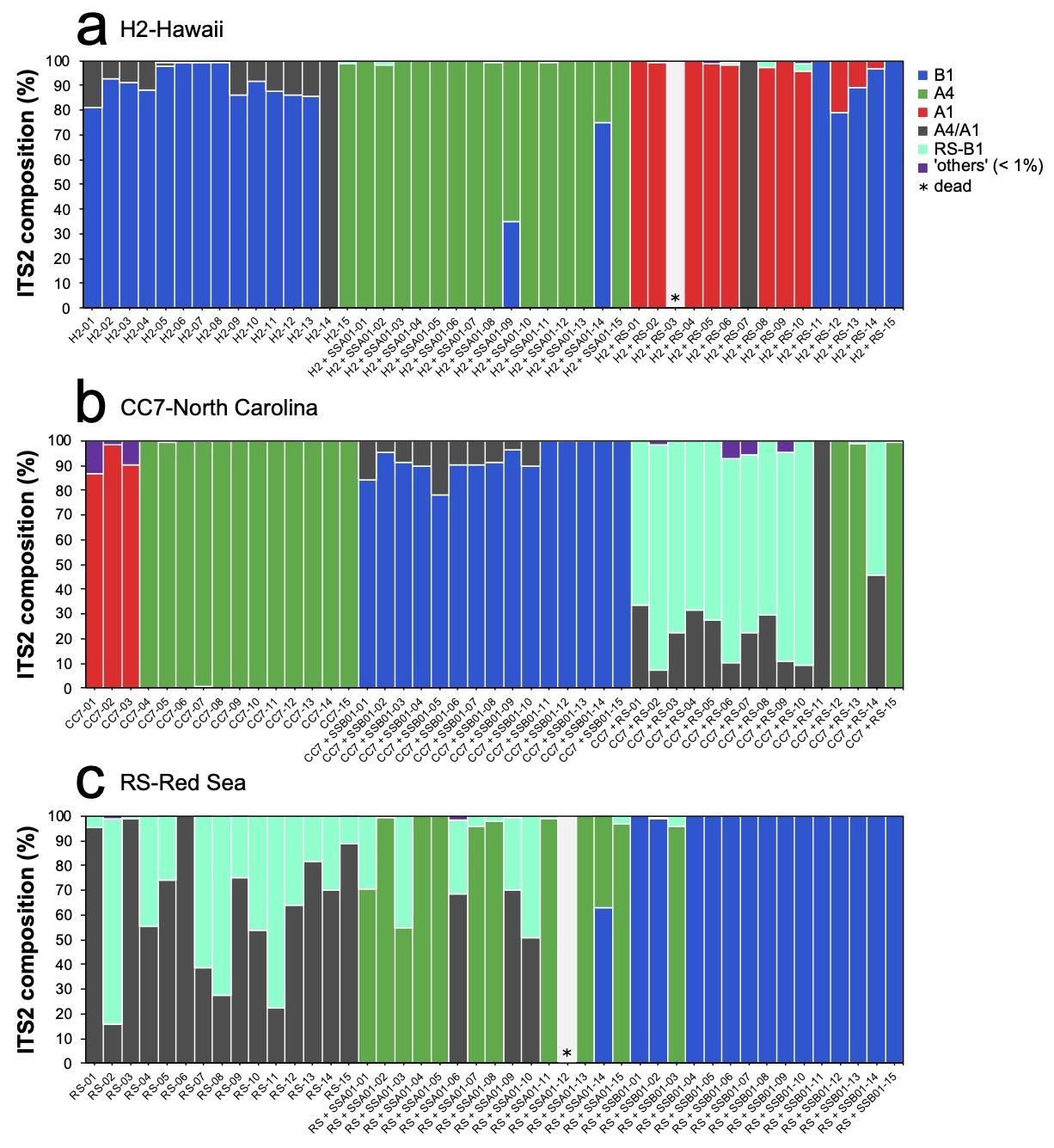


**Figure S2.** ITS2 composition after 6 months of performing initial inoculations. Putative taxa were identified based on the majority sequences as determined by SymPortal. B1 taxa from the Red Sea isolate are designated as RS-B1. Sequences belonging to other taxa and for which abundance was below 1 % were classified as ‘others’


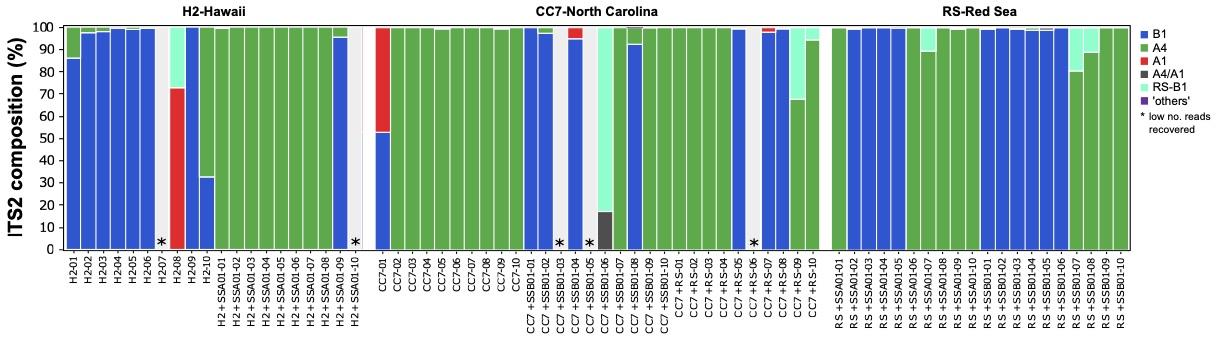


**Figure S3.** ITS2 composition after 1 year of performing initial inoculations. Putative taxa were identified based on the majority sequences as determined by SymPortal. B1 taxa from the Red Sea isolate are designated as RS-B1. Sequences belonging to other taxa and for which abundance was below 1 % were classified as ‘others’.


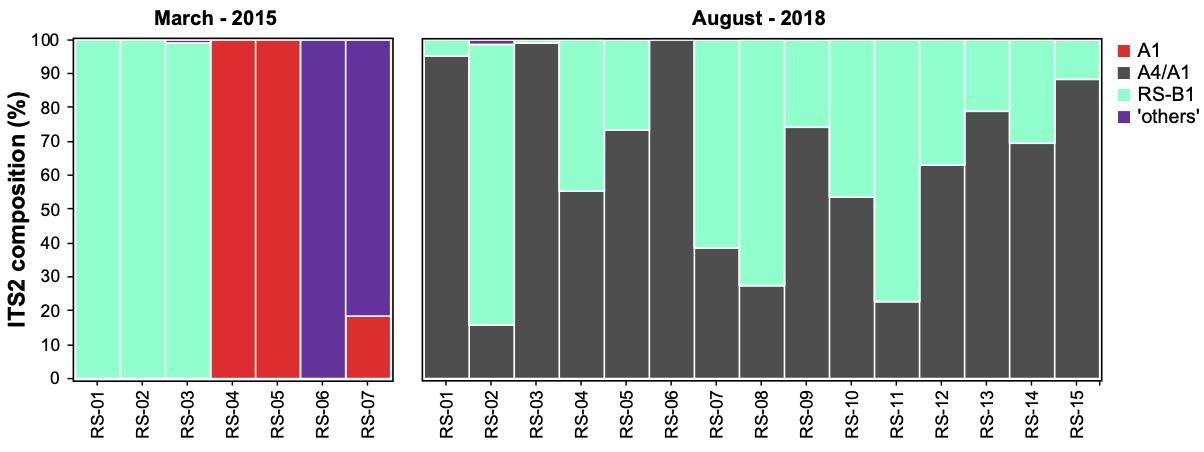


**Figure S4.** Changes in the symbiont composition of Red Sea Aiptasia throughout time. Left panel shows Aiptasia collected in the wild in March 2015 whereas the right panel shows anemone reared under laboratory conditions (sampled in August 2018). Putative taxa were identified based on the majority ITS2 sequences as determined by SymPortal. B1 taxa from the Red Sea isolate are designated as RS-B1. Sequences belonging to clade C and D were classified as ‘others’.

**Figure S5.** Mean (± 1 SE) symbiont cell densities (normalized to protein content) of different Aiptasia holobionts 6 months after initial inoculations. Pairwise comparisons were carried out within each host genotype. Letters above error bars indicate similarities (e.g. AA) or differences (e.g. AB) between host-symbiont combinations, as determined by Tukey’s HSD *post hoc* test (*p* < 0.05). Sample size is indicated in each case.
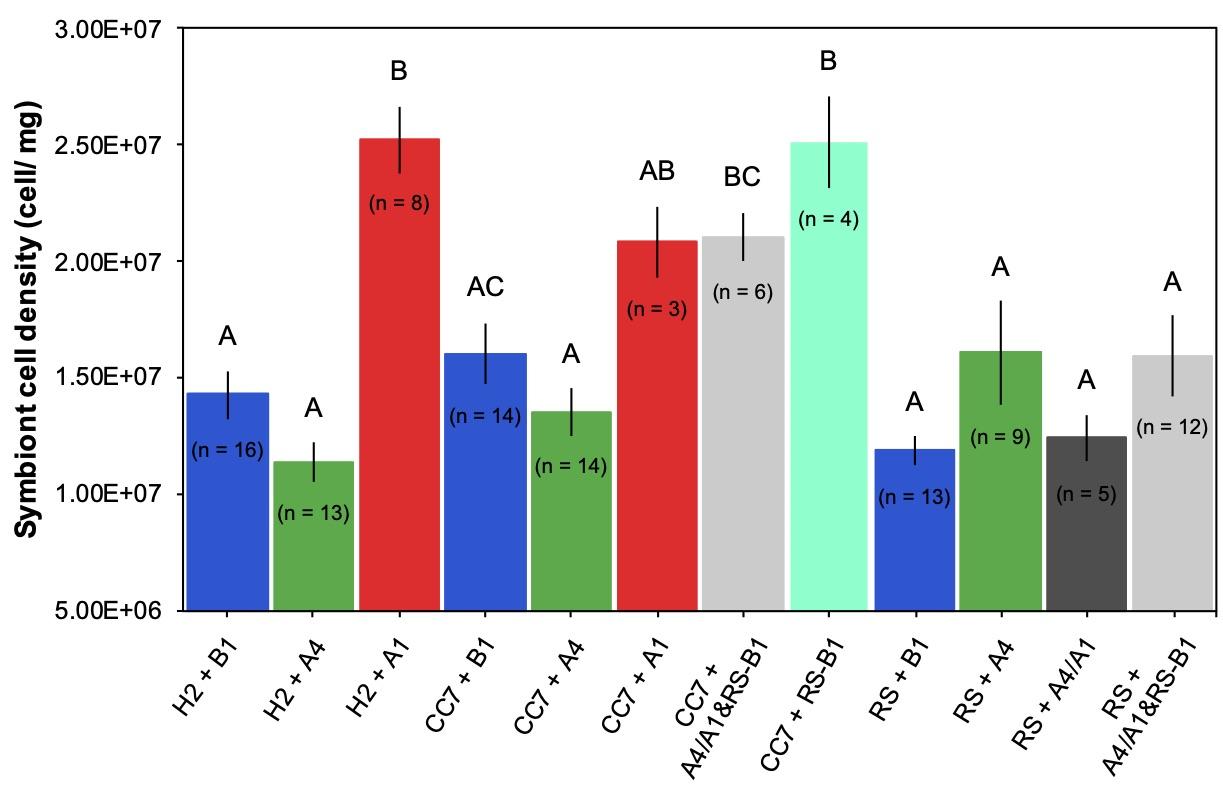


**
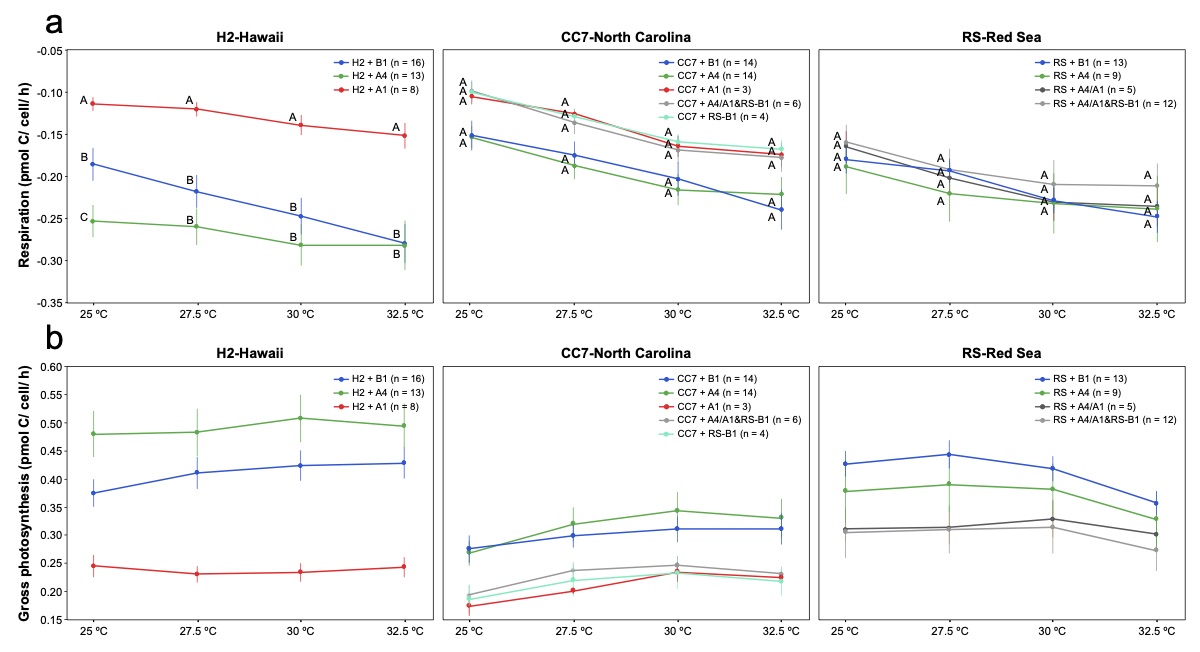
Figure S6.** Mean (± 1 SE) respiration (a) and gross photosynthesis (b) rates across temperature increments for H2, CC7 and RS Aiptasia harboring different symbiont taxa. Letters next to data points indicate similarities (e.g. AA) or differences (e.g. AB) between host-symbiont combinations, as determined by estimated marginal means. Sample size (*n*) for each case is indicated in the legend.

**Table S2.** Summary of results of a linear mixed-models (LMMs) analysis comparing rates of gross photosynthesis and respiration (pmol C/cell/h), and P:R ratios of different Aiptasia-Symbiodiniaceae combinations across temperature increments. Statistically significant *p* values are shown in italic. For each response variable, the significant, highest order terms are denoted in bold.

| Variable |  | Gross photosynthesis | | Respiration | | P:R ratio | |
| --- | --- | --- | --- | --- | --- | --- | --- |
| Transformation |  | Ln | | Ln | | Ln | |
| Model of best fit |  | AR(1) | | AR(1) | | AR(1) | |
| Information criterion |  | AIC = -1518.263  BIC = -1502.102 | | AIC = -1819.626  BIC = -1811.545 | | AIC = -535.715  BIC = -527.634 | |
| Source of variation | Numerator *df* | Denominator *df* | *p* | Denominator *df* | *p* | Denominator *df* | *p* |
| Host | 2 | 116.615 | *0.001*  *F* = 8.002 | 106.631 | 0.092  *F* = 2.441 | 113.331 | *0.003*  *F* = 6.255 |
| Symbiont | 5 | 115.162 | ***< 0.001***  *F* = 7.007 | 106.631 | *0.004*  *F* = 3.732 | 113.331 | *0.006*  *F* = 3.459 |
| Temp | 3 | 37.424 | *0.010*  *F* = 4.319 | 313.638 | *< 0.001*  *F* = 80.651 | 309.711 | *< 0.001*  *F* = 86.573 |
| Host × symbiont | 4 | 116.984 | 0.304  *F* = 1.226 | 313.638 | 0.437  *F* = 0.953 | 113.331 | *0.025*  *F* = 2.900 |
| Host × temp | 6 | 35.735 | ***0.001***  *F* = 4.729 | 106.631 | 0.159  *F* = 1.559 | 309.711 | *< 0.001*  *F* = 4.896 |
| Symbiont × temp | 15 | 38.397 | 0.915  *F* = 0.517 | 313.638 | ***< 0.001***  *F* = 2.862 | 309.711 | *< 0.001*  *F* = 3.562 |
| Host × symbiont × temp | 12 | 35.555 | 0.602  *F* = 0.850 | 313.638 | 0.073  *F* = 1.669 | 309.711 | ***0.004***  *F* = 2.460 |

*df* degrees of freedom, *AIC* (Akaike information criterion), *BIC* (Bayesian information criterion)

**Table S3.** Summary of results of a linear mixed-model (LMM) analysis comparing photochemical efficiencies (*Fv/Fm*) of different Aiptasia-Symbiodiniaceae combinations subjected to long-term temperature stress. Statistically significant *p* values are shown in italic. The significant, highest order terms are denoted in bold.

| Source of variation | Numerator *df* | Denominator *df* | *F* | *p* |
| --- | --- | --- | --- | --- |
| Host | 2 | 90.444 | 20.132 | *< 0.001* |
| Symbiont | 1 | 90.444 | 10.301 | *< 0.05* |
| Day | 28 | 1177.451 | 620.486 | *< 0.001* |
| Host × symbiont | 2 | 90.444 | 4.460 | ***0.014*** |
| Host × day | 56 | 1177.451 | 1.575 | ***0.005*** |
| Symbiont × day | 28 | 1177.451 | 1.739 | ***0.010*** |
| Host × symbiont × day | 56 | 1177.451 | 1.091 | 0.304 |

Data were Ln (*x* +1) transformed

The model of best fit was AR(1)

*AIC* (Akaike information criterion) = -12973.939, *BIC* (Bayesian information criterion) = -12963.226

*df* = degrees of freedom


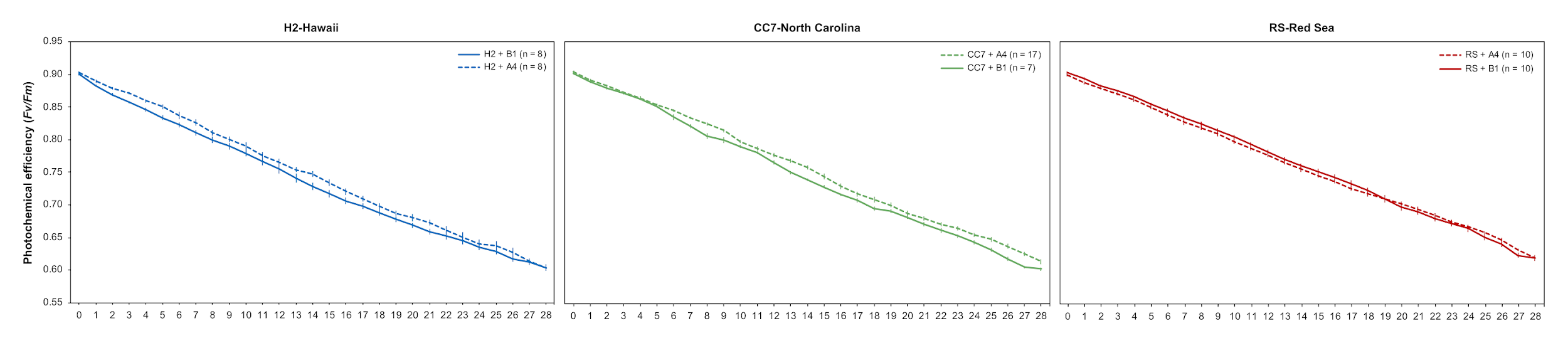


**Figure S7.** Mean (± 1 SE) photochemical efficiencies (*Fv/Fm*) vary between H2, CC7 and RS host-symbiont combinations across time. Sample size (*n*) for each case is indicated in the legend.

**
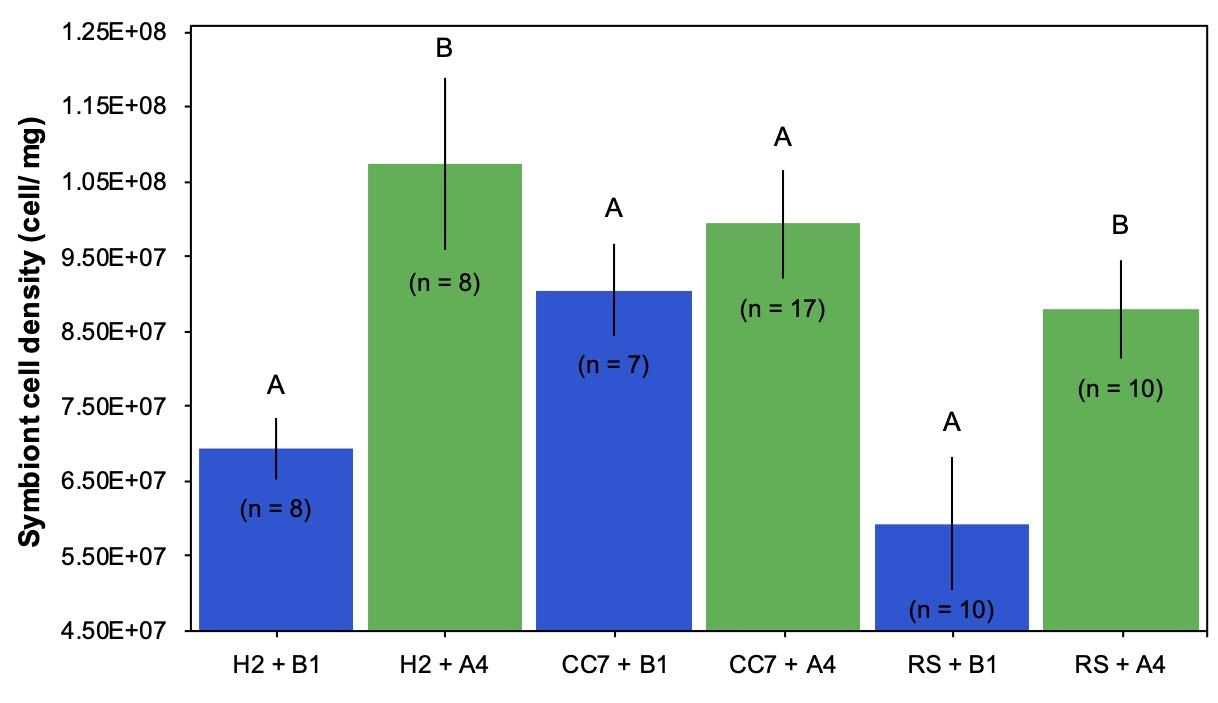
Figure S8.** Mean (± 1 SE) symbiont cell densities (normalized to protein content) of different host-symbiont combinations after 28 consecutive days of heat stress. Pairwise comparisons were carried out within each host genotype. Letters above error bars indicate similarities (e.g. AA) or differences (e.g. AB) between host-symbiont combinations, as determined by Tukey’s HSD *post hoc* test (*p* < 0.05). Sample size is indicated in each case.

**Table S4.** Summary of results for three linear mixed-models (LMMs) comparing the activation energy (eV) associated with rates of gross photosynthesis and respiration, P:R ratios and photochemical efficiencies (*Fv/Fm*) of different Aiptasia-Symbiodiniaceae combinations subjected to heat stress. Statistically significant *p* values are shown in italic. In each case, the significant source of variation is shown in bold.

| Variable |  |  | Gross photosynthesis | Respiration | P:R ratio |  |  | *Fv/Fm* |
| --- | --- | --- | --- | --- | --- | --- | --- | --- |
| Transformation |  |  | Ln (*x* +1+a)^*^ | Ln (*x* +1+a)^*^ | none |  |  | Ln (*x* +1+a)^*^ |
| Information criterion |  |  | AIC = -158.440  BIC = -155.786 | AIC = -127.973  BIC = -125.320 | AIC = -60.303  BIC = -57.649 |  |  | AIC = -238.318  BIC = -236.329 |
| Source of variation | Numerator *df* | Denominator *df* | *p* | *p* | *p* | Numerator *df* | Denominator *df* | *p* |
| Host | 2 | 105 | *< 0.001*  *F* = 37.357 | *< 0.001*  *F* = 13.158 | *< 0.001*  *F* = 9.500 | 2 | 54 | ***0.013***  *F* = 4.730 |
| Symbiont | 5 | 105 | 0.078  *F* = 2.047 | *< 0.001*  *F* = 6.392 | *< 0.001*  *F* = 6.635 | 1 | 54 | 0.399  *F* = 0.723 |
| Host × symbiont | 4 | 105 | ***0.045***  *F* = 2.527 | ***0.041***  *F* = 2.593 | ***0.004***  *F* = 4.135 | 2 | 54 | 0.468  *F* = 0.770 |

*df* degrees of freedom, *AIC* (Akaike information criterion), *BIC* (Bayesian information criterion)

^*^ a is the most negative value in the data to convert all values to positive for Ln transformation

**
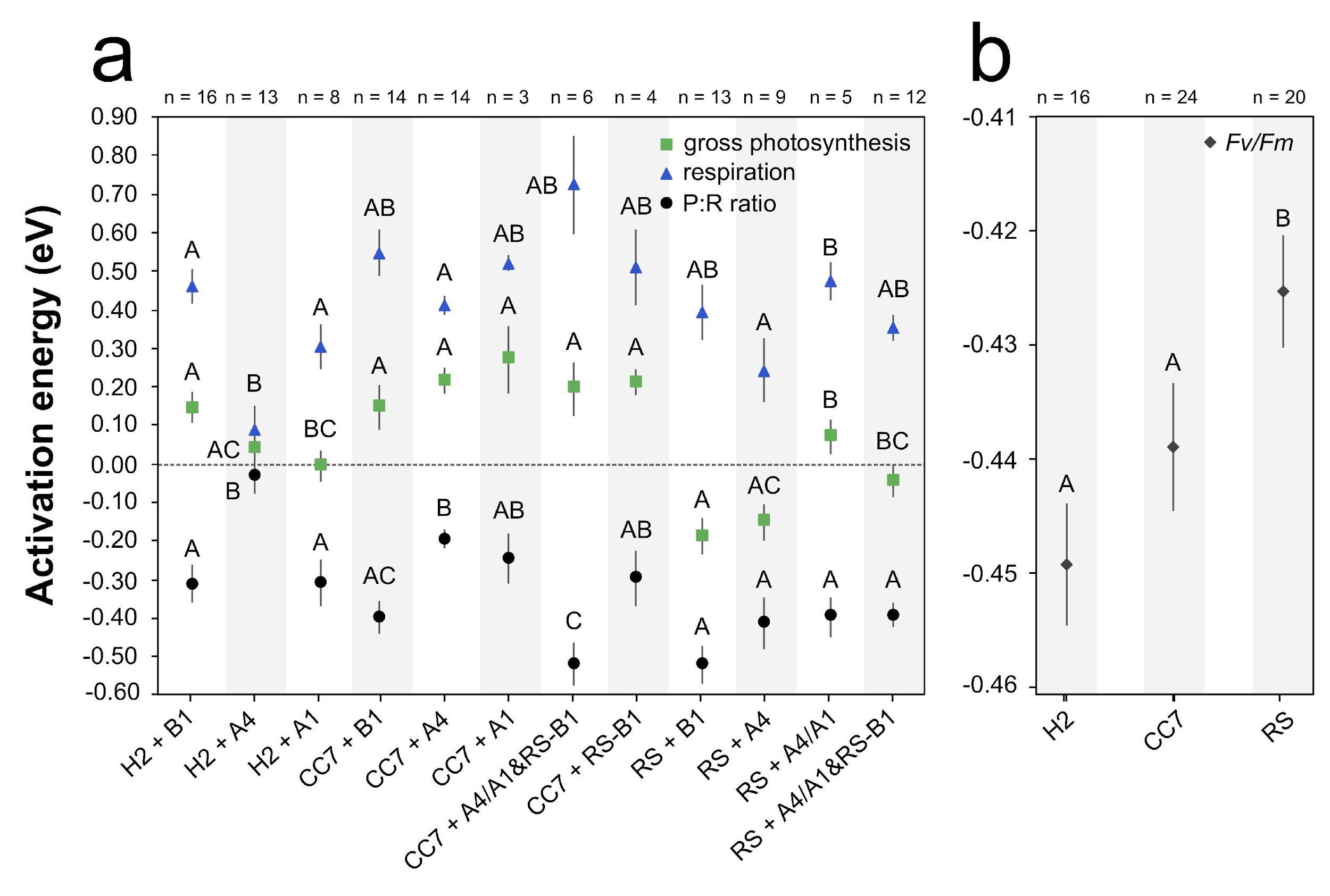
Figure S9.** Mean (± 1 SE) activation energy (eV) associated to **(a)** gross photosynthesis, respiration rates and P:R ratios, and **(b)** photochemical efficiency across different Aiptasia holobionts. Pairwise comparisons were carried out for each response variable and within each host genotype. Letters next to data points indicate similarities (e.g. AA) or differences (e.g. AB) as determined by estimated marginal means. Sample size (*n*) is indicated for each case.
